# Increasing daily feeding occasions in restricted feeding strategies does not improve performance or well being of fattening pigs

**DOI:** 10.1186/1751-0147-50-24

**Published:** 2008-06-24

**Authors:** Eva Persson, Margret Wülbers-Mindermann, Charlotte Berg, Bo Algers

**Affiliations:** 1 Department of Animal Health and Environment, Swedish University of Agricultural Sciences, P.O. Box 234, SE-532 23 Skara, Sweden; 2 Swedish Board of Agriculture, SE-551 82 Jönköping, Sweden

## Abstract

**Background:**

The natural feeding behaviour of the pig is searching for feed by rooting activities throughout the day; self-feeding pigs randomly space their eating and drinking periods throughout the day consuming ten to twelve meals per day. Pigs in conventional fattening pig production are normally fed 2–3 times daily with the feed consumed within 15 minutes. The aim of this study was to determine if more frequent feedings could improve the performance of conventionally kept fattening pigs.

**Methods:**

The experiment was carried out on 360 fattening pigs (27–112 kg live weight), weighed and assigned to pens stratified by weight and sex. Each treatment group consisted of 180 pigs, allocated to 20 pens with nine pigs in each pen. To evaluate how more feeding occasions affects performance and well-being the pigs were divided into two groups and fed three (control group) or nine (treatment group) times daily. The same total amount of liquid feed was fed to each group and the feed ration was correlated to the live weight of the pigs. All weight and slaughter recordings were made individually and recordings of feed consumption were made pen-wise. At slaughter the stomach of each pig was examined for lesions in the pars oesophagea and scored on a scale from 1–6.

**Results:**

Frequent feeding occasions influenced both performance and status of gastric lesions of the pigs adversely. Pigs in the treatment group grew slower compared to pigs in the control group; 697 g/day (± 6.76) versus 804 g/day (± 6.78) (P < 0.001) with no difference in within-pen variation. There was also a lower prevalence of gastric lesions within pigs in the control group (2.4 (± 0.12) compared to 3.0 (± 0.12) (P < 0.01)). There was a positive correlation between gastric lesions in the treatment group and daily weight gain (r = 0.19; P < 0.01).

**Conclusion:**

Increased daily feeding occasions among group housed pigs resulted in a poorer daily weight gain and increased mean gastric lesion score as compared with pigs fed three times daily. This may be a consequence of more frequently occurring competition for feed in the treatment group. The present study does not support increased daily feeding occasions in fattening pigs.

## Background

The natural feeding behaviour of the pig is searching for feed by rooting activities throughout the day and Stolba and Wood-Gush [1] reported that pigs living in a semi-natural environment (including grass and woodland) spent 20% rooting and 30% grazing during daylight. Self-feeding pigs randomly space their eating and drinking periods throughout the day and ad libitum fed pigs eat ten to twelve meals per day [2]. In contrast, pigs in conventional indoor fattening pig production are normally fed 2–3 times daily whereby the feed is consumed within 15 minutes after feeding. This corresponds to approximately 5% of the time that pigs kept in a semi-natural environment typically spend foraging.

Feeding behaviour is stimulated by the sight of other pigs feeding [3] and group housed pigs consume more feed than individually kept ones. Knowledge about the biological needs of the pig makes it easier to improve the development of housing systems in fattening pig production. Increasing the feeding frequency is only one management factor which might facilitate the performance of the natural behaviour. Physiological parameters could show a positive correlation between feeding frequency and digestion [4]. Frequent small meals have also been reported to lead to a higher lean tissue content of the carcass [5,6].

The aim of the present study was primarily to evaluate the effect of increased daily feeding occasions on production performance and the occurrence of gastric lesions. A secondary endpoint was to determine whether increased feeding occasions could improve the pigs' welfare using these outcome measures as indicators.

## Methods

### Experimental design

A total of 360 crossbred pigs ((Swedish Landrace × Swedish Yorkshire) × Hampshire) were delivered from four commercial piglet herds and raised from 27 to 112 kg live weight at the Swedish University of Agricultural Sciences' Experimental station in Western Sweden. The pigs were given individual ear tags and were randomly allocated to two treatment groups according to sex, weight and herd of origin. Each treatment group consisted of 180 pigs, allocated to 20 pens with nine pigs in each pen. The pens measured 3.0 × 1.8 m with an additional concrete and slatted floor (1.50 × 1.20 m) in the dunging area, giving a total area of 0.8 m^2 ^per pig [7]. The feeding trough was 3.0 m, giving enough feeding space for all pigs to access the trough [7]. Approximately 2 kg of chopped straw was scattered on the floor once daily after cleaning. The treatment groups varied by frequency of feeding; the control group was fed three times daily and the treatment group was fed nine times daily. The experimental design and feeding times are shown in Table 1.

**Table 1:** The experimental design

	Control group	Treatment group
Feedings, times daily	3	9
No. of Animals	180	180
Feeding time		
Morning, h	07.00	07.00, 07.45, 08.30
Lunch, h	13.00	13.00, 13.45, 14.30
Evening, h	20.00	20.00, 20.45, 21.30

### Feed rations and nutrient content

All pens (each containing nine pigs) received the same type and amount of liquid feed, based on a compound feed for fattening pigs, mixed with whey and water (Table 2). The ratio between feed and whey plus water was 1:3. In total, 19% of the energy in the liquid feed mixture originated from whey. The crude protein content in the compound feed was analysed to be 180 g/kg feed with an energy level of 12.6 ME MJ/kg. The analysed lysine level was 10.9 g/kg feed and the total amount of methionine and threonine were calculated to be 34% and 59% of the dietary lysine level, respectively. The pigs were fed restrictedly in a trough according to the standard feeding regime for growing pigs in Sweden with a daily feed ratio of metabolisable energy (ME) of 16.5, 19.0, 24.1, 29.0 and 34.1 MJ at 25, 30, 40, 50, 60 kg live weight and thereafter to slaughter, respectively [8].

**Table 2:** Nutrient content in the compound feed and composition of the liquid feed mixture

Chemical composition	Compound feed^1^	Liquid mixture^2^
Water, %	12.2	73.1
Energy, ME MJ/kg	12.6	3.9
Crude protein, g/kg	180	48.0
Lysine, g/kg	10.9	2.9
Methionine, g/kg		1.0
Threonine, g/kg		1.7
Methionine/Lysine, %		34
Threonine/Lysine, %		59
Calcium, g/kg	7.30	2.3
Phosphorus, g/kg	5.70	1.8

^1. ^Analysed nutrient content.

^2. ^Calculated nutrient content in the liquid mixture based on analysed nutrient content in the compound feed and declared nutrient value of the whey.

All pigs were weighed individually every second week and the feed ratio was calculated from the average live weight of the pigs in each pen. The "Big Dutchman" feeding computer system dispensed feed and recorded feed intake and increased feed intake daily from a predicted weight gain which was corrected after every weighing occasion. Daily, weekly and total feed consumption and feed conversion was recorded automatically and calculated per pen.

### Slaughtering of pigs

Slaughter was performed at approximately 112 kg live weight at a commercial abattoir located 30 minutes drive from the experimental farm. During the slaughtering period the pigs were weighed every week. When the third last pig in every pen reached 112 kg live weight, all three were sent for slaughter. At the abattoir carcass weight, lean meat percentage, fat measurements and a health control of inner organs was recorded individually for every pig according to the usual routine at slaughter.

The stomach of every pig was opened along the greater curvature, emptied and examined for lesions in the pars oesophagea. The appearance of the mucous membrane in the pars oesophagea was analysed according to a scale (Table 3) developed by Simonsson [9]. Due to practical implications at slaughter, only stomachs from 177 pigs in the control group and 175 pigs in the treatment group could be used in the analysis of data.

**Table 3:** Scale for analysing lesions in the pars oesophagea (Simonsson, 1977)

Score	Designation	Description of alterations to mucous membrane
1	Normal	Cutaneous membrane was thinly-folded with a with glistering surface
2	Slight parakeratosis	Patchy, thickened and yellowish discolouration of membrane
3	Moderate parakeratosis	More severe than the previous case
4	Severe parakeratosis	Whole membrane severely papillary thickened with a dirty yellow or grey discolouration
5	Erosions	Initial stages of epithelial losses
6	Ulcers	Ulcerated mucous membrane, with or without bleeding

### Statistical methods

Data were analysed using the GLM and Pearson's Partial correlation procedure [10]. The effect of feeding occasions on production performance and prevalence of gastric lesions were studied in a random design. The statistical model included the effect of feeding occasion and sex as fixed factors and initial weight as a covariate. Interactions between fixed factors were tested and included when significant (P < 0.05). All results are presented as least square means with standard errors. The individual pig was used as the experimental unit for all traits except for feed consumption, feed conversion ratio and standard deviation within pen for several traits, where the pen was the experimental unit.

## Results

One pig in the control group was excluded from the trial due to illness. No significant interactions between treatment and sex could be found. Results of production traits are shown in table 4. The average initial weight was 27.2 kg for both treatment groups. Pigs in the control group had a higher final weight (113.6 (± 0.29) vs. 112.6 kg (± 0.29)) (P < 0.05) and carcass weight (87.3 (± 0.26) vs. 86.4 kg (± 0.26)) (P < 0.01) than pigs in the treatment group.

**Table 4:** Effects of feeding frequency on performance and gastric lesions for control and treatment group (least square means ± standard errors)

	Control group	Treatment group	Sign.
No. of animals	179	180	
Initial weight, kg	27.2	27.2	
Final weight, kg	113.6 (± 0.29)	112.6 (± 0.29)	*
Carcass weight, kg	87.3 (± 0.26)	86.4 (± 0.26)	*
Daily weight gain, g			
Entire fattening period	804 (± 6.78)	697 (± 6.76)	***
Start – 60 kg	788 (± 7.98)	638 (± 7.96)	***
60 kg – slaughter	815 (± 8.22)	744 (± 8.20)	***
Feeding period, days	109 (± 0.88)	125 (± 0.88)	***
Lean meat content, %	57.2 (± 0.20)	56.6 (± 0.20)	0.06
Gastric lesions, score	2.4 (± 0.12)	3.0 (± 0.12)	**

Level of significance: n.s. = p > 0.05; * = p < 0.05; ** = p < 0.010; *** = p < 0.001.

^1 ^MJ = Mega Joule in Metabolisable energy.

### Daily weight gain

**Figure 1:**
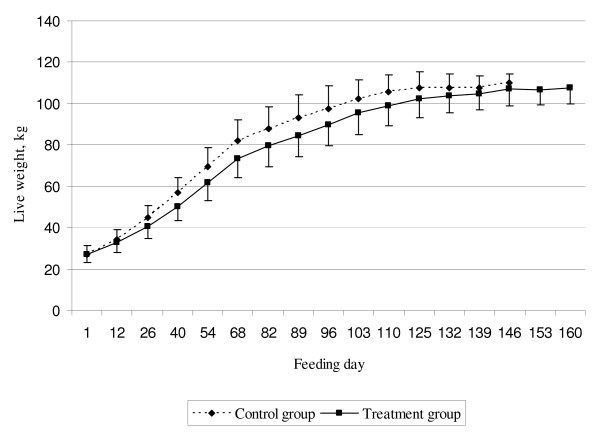
Development of live weight gain during the fattening period.

The pigs in the treatment group, with nine feedings a day, had a lower daily weight gain during the entire fattening period (697 g (± 6.76)) compared to pigs the control group (804 g (± 6.78)) (P < 0.001). The difference in daily weight gain was larger during the period up to 60 kg live weight (150 g) (638 g (± 7.96) vs. 788 g (± 7.98)) than during the period from 60 kg live weight to slaughter (71 g) (744 g (± 8.20) vs. 815 g (± 8.22)) (P < 0.001) (Figure 1 and Table 4). The rearing period was 109 days for the control group and 125 days for the treatment group (P < 0.001). Pigs in the control group reached slaughter weight 16 days earlier and with a tendency to a higher lean meat content (57.2% (± 0.20) vs. 56.6% (± 0.20)) (P = 0.06) than pigs in the treatment group (Table 4). Male pigs had a significantly higher daily weight gain (783 g vs. 718 g) (P < 0.001) during the entire fattening period while the female pigs had 0.8% higher lean meat content in the carcass (57.3% vs. 56.5%) (P < 0.01).

### Gastric lesions

**Figure 2:**
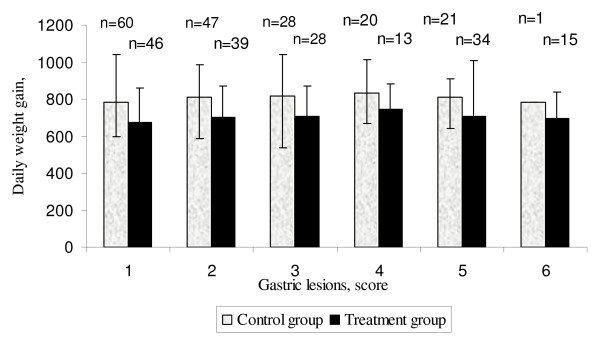
Correlation between daily weight gain and score of gastric lesions (n = indicates the number of pigs in each category).

Gastric lesions were found in 61% of the pigs, scoring 2–6 according to Simonsson (Table 3). In the control group and treatment group, 40% and 51.4% of the pigs respectively, had gastric lesion scores between 3 (moderate parakeratosis) and 6 (ulcers) [9]. Furthermore, pigs in the treatment group had a higher mean gastric lesion score (3.0 vs. 2.4) (P < 0.001) compared to pigs in the control group (Table 4). However, there was no difference between sexes; both male (2.7) and female (2.7) pigs proved to have equal mean gastric lesion scores. There was a significant correlation between daily weight gain and gastric lesion score r = 0.19, (P < 0.01) (Figure 2).

### Within pen variation

There was no significant difference in within pen variation for daily weight gain between treatment groups. The within pen variation in daily weight gain for the entire period was 96 g (± 5.94) for the control group and 92 g (± 5.94) for the treatment group. There was no difference between the first period (120 g (± 6.46) vs. 103 g (± 6.46)) and the last period (165 g (± 25.34) vs. 121 g (± 25.34)) of the fattening period (Table 5). Within pen variation for feeding days was 13 (± 0.73) for the control group and 16 (± 0.73) for the treatment group respectively (P < 0.05). The treatment group also showed greater variation in lean meat content (2.9% (± 0.18) vs. 2.4% (± 0.18), P < 0.05) and gastric lesion score (1.7 (± 0.07) vs. 1.4 (± 0.07), P < 0.05).

**Table 5:** Effects of feeding frequency on within pen variation in performance (least square means ± standard errors)

Within pen variation	Control group	Treatment group	Sign.
Daily weight gain, g			
Entire period, g	96 (± 5.94)	92 (± 5.94)	n.s.
Start – 60 kg	120 (± 6.46)	103 (± 6.46)	n.s.
60 kg – slaughter	165 (± 25.34)	121 (± 25.34)	n.s.
Feeding period, days	13 (± 0.73)	16 (± 0.73)	*
Lean meat content, %	2.4 (± 0.18)	2.9 (± 0.18)	*
Gastric lesions, score	1.4 (± 0.07)	1.7 (± 0.07)	*

Level of significance: n.s. = p > 0.05; * = p < 0.05; ** = p < 0.010; *** = p < 0.001.

## Discussion

Today almost 70% of all fattening pigs in Sweden are fed liquid feed. It is possible to increase the feeding occasions without increasing work load. Many farmers claim that increasing the feeding occasions from twice to three or four times a day improves the stable environment. In this study the aim was to attempt to reproduce the natural feeding pattern of pigs by more frequent feeding. We arranged the nine feedings around clusters of three for practical and feed hygiene reasons, instead of equally distributed over the day. This is a potential limitation of the study but we believe that it would be unlikely to change the results of the study. The only variable was the frequency of feeding in the control and treatment groups which means that the difference in daily weight gain performance has to be explained by competition amongst the pigs in the pen and a different feed intake pattern. The results in the present study indicate that increased daily feeding occasions resulted in poorer performance, measured as daily weight gain (697 g/day), a tendency to a lower lean meat content (56.6%) and a higher prevalence of gastric lesions (score 3.0), compared with pigs fed only three times daily (804 g/day, 57.2% and score 2.4).

Results from both Ruckebusch and Bueno and Botermans and co-workers have shown beneficial effects on growth with more efficient digestion of nutrients when individually housed pigs were fed many meals of limited size [2,11]. Also, de Haer and de Vries showed that many, relatively small, meals a day had a positive influence on the digestibility of the feed among group housed pigs [5]. Group housed pigs establish a different feed intake pattern caused by social interactions; a pig in a group has to eat quickly to get enough feed because of competition with other pigs. It is possible that each feeding occasion in our study offered too small amounts of feed to satisfy the hunger of the pigs and hence gave different results from these earlier studies.

Competition during feeding is one of several factors that can give a lower daily weight gain amongst pigs. According to Baxter [12] ninety percent of all aggressive interactions between pigs occur during feeding. Competition includes fighting for both rank within the group at feeding and for the limited space in the pen. Without competition pigs within the pen achieve the same daily weight gain according to their body weight but when the feeding space is limited competition is most clearly evident for the smallest pigs [13]. A highly competitive feeding environment causes a larger variation in daily weight gain and carcass meat percentage [14,15].

Competition for feed within the pen could not fully explain the lower daily weight gain observed for the treatment group. There was no significant difference in within-pen variation for daily weight gain between the control and treatment group (96 g/day vs. 92 g/day) and the individual feeding space of 33 cm per pig is considered adequate to allow all pigs access to the trough (and is the Swedish Animal Welfare Legislation Standard). Recently however, Turner and co-workers have suggested 42.5 cm per pig at the trough to allow all pigs to eat simultaneously without depression of growth [16]. The finding that no greater variation occurred when the feed was limited might be a consequence of the increased feeding frequency allowing all pigs to eat equivalent small servings. Insufficient servings at each feeding occasion might also have left pigs in the treatment group hungry and created a stressful situation. Indeed, in work already published from this study, Hessel and co-workers [17] found that smaller servings at every feeding occasion resulted in a more competitive situation as they found that more frequently fed pigs displayed more aggressive actions, achieved higher scores for skin lesions and had a tendency to belly-nose for longer time periods when compared with pigs fed less frequently.

Batterham and Bayley [18] reported that feed intake patterns influence fat and lean growth in pigs through an effect on utilisation of nutrients. Both de Haer and de Vries and Ramaekers and co-workers [5,6] showed that frequent small feedings led to a higher lean meat content of the carcass but in the present study there was a tendency for a higher lean meat content for pigs in the control group (57.2 vs. 56.6%). Increased competition/stress may also explain the discrepancy between our study and the above findings.

Gastric lesions are associated with modern fattening pig production and the best managed herds, with the best production performances, often have the highest prevalence of ulcers [19]. In our study 61% of the pigs had significant gastric lesions. In the present study the increased daily feeding occasions resulted in a significantly higher gastric lesion score (3.0 vs. 2.4) compared with pigs in the control group. This may be the result of a more stressful environment that triggered increased production of cortisol from adrenal cortex [20] and hence increased production of gastric acid. The treatment group also showed a larger variation in gastric lesion score within pen compared with the control group (1.7 vs. 1.4). Hessing and co-workers reported a higher sensitivity for gastric lesions amongst middle ranked pigs compared to high and low ranked pigs [21]. Their finding that pigs vary in their sensitivity to gastric lesions was dependent upon rank may explain our result of greater variation in gastric lesion score within the treatment group.

The correlation between daily weight gain and gastric lesion score was weak (r = 0.19) but still statistically significant, probably due to the large number of observations (352). Extensive gastric lesions will decrease daily weight gain [22] but mild to moderate gastric lesions have been reported not to have an adverse effect on growth rate [23].

## Conclusion

Our results indicate that feeding pigs nine times daily was more stressful to the pigs than feeding three times daily, hampering growth and inducing gastric lesions. This may be a consequence of more frequently occurring competition for feed in the treatment group. The present study does not support the use of increased feeding occasions in fattening pigs.

## Competing interests

The authors declare that they have no competing interests.

## Authors' contributions

EP and MWM designed the experiment and collected data, EP compiled the results and drafted the manuscript, CB and BA initiated the study, participated in the design and coordinated the study. All authors read and approved the manuscript.
